# Revised sample preparation for the analysis of oxysterols by enzyme-assisted derivatisation for sterol analysis (EADSA)

**DOI:** 10.1007/s00216-015-8609-2

**Published:** 2015-03-22

**Authors:** Peter J. Crick, T. William Bentley, Yuqin Wang, William J. Griffiths

**Affiliations:** 1 College of Medicine, Swansea University, Grove Building, Singleton Park, Swansea, SA2 8PP UK; 2 Present Address: Medical Parasitology and Infection Biology, Swiss Tropical and Public Health Institute, Socinstrasse 57, 4002 Basel, Switzerland

**Keywords:** Sample preparation, Solid-phase extraction, Lipidomics, Cholesterol, Oxysterol, Derivatisation, Liquid chromatography, Mass spectrometry

## Abstract

Sterols, and specifically oxysterols, play important roles in the biosynthesis of bile acids and steroid hormones as well as possessing biological activities in their own right. Analysis of oxysterols is complicated due to their low abundance in biological systems and poor ionisation characteristics in mass spectrometry. Over the past decade, we have developed a liquid chromatography–mass spectrometry method termed enzyme-assisted derivatisation for sterol analysis (EADSA). Our derivatisation procedure relies on two solid-phase extraction steps to (i) separate cholesterol from oxysterols and (ii) remove excess derivatisation reagents. Recent inter-batch variation in C18 reversed-phase cartridges has led us to experiment with alternative columns. Here, we present our findings and report an improved sample preparation procedure using polymeric hydrophilic–lipophilic balanced reversed-phase cartridges.

**Electronic supplementary material:**

The online version of this article (doi:10.1007/s00216-015-8609-2) contains supplementary material, which is available to authorized users.

## Introduction

Oxysterols are oxidised forms of cholesterol incorporating one or more hydroxyl, carbonyl, epoxide or carboxylic acid group onto the cholesterol skeleton. Historically, oxysterols have been known as intermediates in the biosynthesis of bile acids and as important transport forms of cholesterol. However, in recent years, interest in these compounds has grown as they have been shown to have important signalling roles, for example, as agonists to the liver X receptors (LXRs) and the G protein-coupled receptor Epstein-Barr virus-induced receptor 2 (EBI2, GPR183). Some studies have also shown potential roles for oxysterols in the progression of neurodegenerative diseases, the formation of atherosclerotic plaques and as biomarkers for oxidative stress [1].

Biosynthesis of oxysterols from cholesterol is catalysed by a number of sterol hydroxylases, often members of the cytochrome P450 (CYP) family. For example, CYP7A1 converts cholesterol to cholest-5-ene-3β,7α-diol (7α-hydroxycholesterol, 7α-HC) in the first step of the neutral pathway of bile acid biosynthesis. Similarly, CYP27A1 catalyses the formation of cholest-5-ene-3β,(25R)26-diol ((25*R*)26-hydroxycholesterol, 26-HC) in the first step of the acidic pathway. Note, we use the systematic nomenclature where addition of a hydroxyl group to the terminal carbon of the cholesterol side-chain introducing R stereochemistry at C-25 is said to be at C-26 [2].

The low levels of endogenous oxysterols (pg/mL to ng/mL in human plasma) coupled with a lack of chromophore and poor ionisation characteristics make analysis challenging [1]. Gas chromatography (GC)–mass spectrometry (MS) following sample extraction, hydrolysis and derivatisation has long been considered the ‘gold standard’ analytical method [3]. Recently, several liquid chromatography (LC)-MS methods have been reported both with and without prior derivatisation to improve ionisation [1, 4, 5].

In any method of oxysterol analysis products formed by non-enzymatic autoxidation of cholesterol during sample storage, handling and workup can complicate reliable identification and quantification. Methods used to avoid artefacts derived from cholesterol include the use of antioxidants such as butylated hydroxytoluene (BHT), strict exclusion of atmospheric oxygen using an inert gas such as argon and reducing photo-induced oxidation by handling samples in the dark or in low light. An alternative strategy, which we favour, relies on prompt separation of cholesterol from oxysterols at the earliest possible point of the sample workup using solid-phase extraction (SPE). After separation of oxysterols from cholesterol by SPE-1, we carry out an enzymatic oxidation of the characteristic oxysterol 3β-hydroxy-Δ-5 group to the corresponding 3-oxo-Δ-4 moiety, followed by a ‘click-chemistry’ reaction with the Girard P (GP) hydrazine reagent to introduce a permanently charged quaternary ammonium group (see Electronic Supplementary Material (ESM) Fig. S1). This greatly enhances signal in electrospray ionisation (ESI), by a factor of about 10^2^–10^3^, and improves solubility in reversed-phase solvents to aid LC separation of oxysterols. In addition, the GP-derivatised sterols give a characteristic fragmentation pattern upon tandem MS (MS^n^) analysis. We have termed this method enzyme-assisted derivatisation for sterol analysis (EADSA) [5].

After derivatisation, a second SPE step (i.e. SPE-2, ESM Fig. S2) is necessary to remove excess reagent before analysis. We have used a 200-mg Waters Sep-Pak tC18 reversed-phase cartridge with a method optimised for the recovery of oxysterols with a side-chain hydroxyl group (e.g. 22*R*-, 24*S*-, 25- and 26-HC) and for cholestenoic acids (e.g. 3β-hydroxycholest-5-enoic acid and 3β,7α-dihydroxycholest-5-enoic acid). However, recent experience has shown considerable batch-to-batch variation in the performance of this cartridge. Here, we describe our efforts to re-optimise our SPE procedures using a number of different C18 and a polymeric reversed-phase sorbents.

## Experimental section

### Materials

Absolute ethanol and HPLC grade solvents were from Fisher Scientific (Loughborough, UK). Acetic acid was AnalaR NORMAPUR grade (BDH, VWR, Lutterworth, UK) and formic acid was from Sigma-Aldrich (Dorset, UK). Authentic sterol standards were from Avanti Polar Lipids (Alabama, USA) or Sigma-Aldrich. Cholesterol oxidase was from Sigma-Aldrich and the GP reagent was from TCI Europe (Oxford, UK). The plasma sample used was from Sigma-Aldrich. Certified Sep-Pak tC18 200-mg cartridges and Oasis HLB 60-mg cartridges were generously donated by Waters (Elstree, UK). Telos C18 with and without endcapping (Kinesis), Isolute C18 and Hypersep C18 (ThermoFisher) were generous gifts from the manufacturers.

### Methods

The derivatisation and LC-MS^n^ methods (ESM Fig. S1 and S2) are described in detail in Griffiths et al. [5].

## Results

### Inter-batch variation in Waters Sep-Pak tC18 cartridges

When analysing a plasma sample as part of a recent study, we observed very low intensity signals for a range of analytes including our internal standard, 24*R*/*S*-[25,26,26,26,27,27,27-^2^H_7_]hydroxycholesterol ([^2^H_7_]24*R*/*S*-HC). To try and identify the problem, we initially used a simple mixture of synthetic standards to very approximately mimic the steroid and sterol content of plasma. This was made up of 5 μg of [^2^H_7_]24*R*/*S*-HC, cholest-4-en-3-one and dehydroepiandrosterone sulphate (DHEAS). These compounds cover a wide range of hydrophobicity and, therefore, have different profiles on reversed-phase SPE and LC columns.

**Fig. 1:**
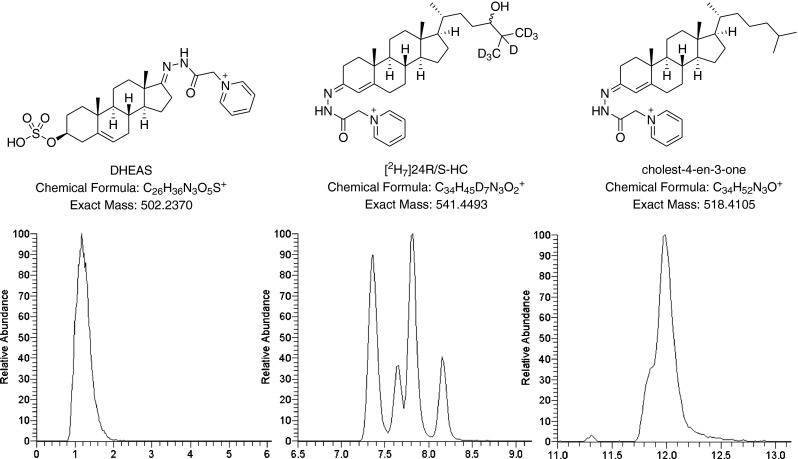
Structures and representative chromatograms of synthetic standards after derivatisation by EADSA. Retention times: DHEAS, 1.15 min; [^2^H_7_]24*R*/*S*-HC, 7.82 min; cholest-4-en-3-one, 11.99 min. [^2^H_7_]24*R*/*S*-HC gives four peaks as each of the *R* and *S* isomers give *syn* and *anti* conformers upon EADSA. SPE-2 was from batch C

Firstly, the mixture of standards was subjected to our established conditions for EADSA and analysed by LC-MS^n^ on the LTQ-Orbitrap Velos. However, as we were analysing a mixture of standards rather than biological samples, the removal of cholesterol by SPE-1 was not deemed necessary. Only the SPE-2 step for the removal of excess derivatisation reagent was carried out. The inclusion of cholest-4-en-3-one was used to control for any problems with the cholesterol oxidase step as cholest-4-en-3-one does not require enzymatic oxidation before derivatisation with the GP reagent. DHEAS is very polar and when derivatised with the GP reagent elutes after about 1 min in our LC gradient, while cholest-4-en-3-one elutes after about 12 min. [^2^H_7_]24*R*/*S*-HC is our usual internal standard and is representative of side-chain hydroxycholesterols, eluting from the LC at about 8 min. Shown in Fig. 1 are reconstructed ion chromatograms (RICs) for these standards recorded earlier under optimal conditions. In our initial study, we replaced all materials with new batches and we were able to rule out problems with solvent purity, reagent stability and enzyme activity as the reason for low signal intensity. However, we observed a marked difference between two batches of Sep-Pak tC18 200-mg cartridges employed for SPE-2. Very poor recovery of [^2^H_7_]24*R*/*S*-HC was observed when using a recent batch, 011133059D (batch D), compared to an older one, 011032331C (batch C) (ESM Figure S3a). Similarly, there was no measurable recovery of cholest-4-en-3-one when batch D was employed (ESM Figure S3b). However, the much less hydrophobic DHEAS eluted well from both batches of cartridge (ESM Figure S3c). While the cause of this inter-batch variation was not immediately clear, it is probable that a cation exchange mechanism is also operating in the D-batch rather than solely reversed-phase interactions as in the C-batch. While GP-derivatised DHEAS in aqueous alcohol is zwitterionic but electrically neutral, sterols and oxysterols are cationic and thus retained by any cation exchange interactions on the D-batch of SPE-2. Consistent with this hypothesis, we found that elution with a more polar solvent (ethanol) had no effect on the outcome of the experiment.

### Other C18 cartridges

In an attempt to find an alternative cartridge suitable for our SPE-2 step, we tested C18 columns from Biotage, Kinesis and ThermoFisher using our mixture of standards. We found that the zwitterionic neutral analyte DHEAS eluted well from all of the cartridges with very little variation in the peak area (ESM Figure S4a). However, [^2^H_7_]24*R*/*S*-HC only eluted satisfactorily from the Sep-Pak tC18 column from batch C, with the other sorbents retaining this analyte (ESM Fig. S4b). The most hydrophobic compound, cholest-4-en-3-one, was not found to elute from any cartridges other than Sep-Pak tC18 batch C (ESM Fig. S4c). This data indicates that like Sep-Pak tC18 batch D, but in contrast to batch C, the other C18 columns retain cationic GP-derivatised analytes.

### Oasis HLB cartridges

As we were unable to obtain satisfactory results with any of the C18 cartridges tested, other than Sep-Pak tC18 batch C, which is no longer commercially available, we next turned our attention to the polymeric hydrophilic–lipophilic balanced reversed-phase Oasis HLB column (ESM Fig. S5). The manufacturer recommends using approximately one third of the sorbent mass compared with an equivalent C18 cartridge. We used the 60-mg Oasis HLB column (cf. 200-mg tC18 Sep-Pak normally used for SPE-2) but maintained the same solvent volumes. With this method, the recoveries using the Oasis HLB cartridge of both DHEAS and [^2^H_7_]24*R*/*S*-HC were as good as when using Sep-Pak tC18 batch C (ESM Fig. S6a and S6b). Surprisingly, the most hydrophobic analyte (cholest-4-en-3-one) gave about a 50-fold larger peak area when using the polymeric cartridge compared to the C18 batch C (ESM Fig. S6c), suggesting that the Oasis HLB sorbent may be particularly useful for the recovery of hydrophobic analytes.

### Testing with a biological sample

**Fig. 2:**
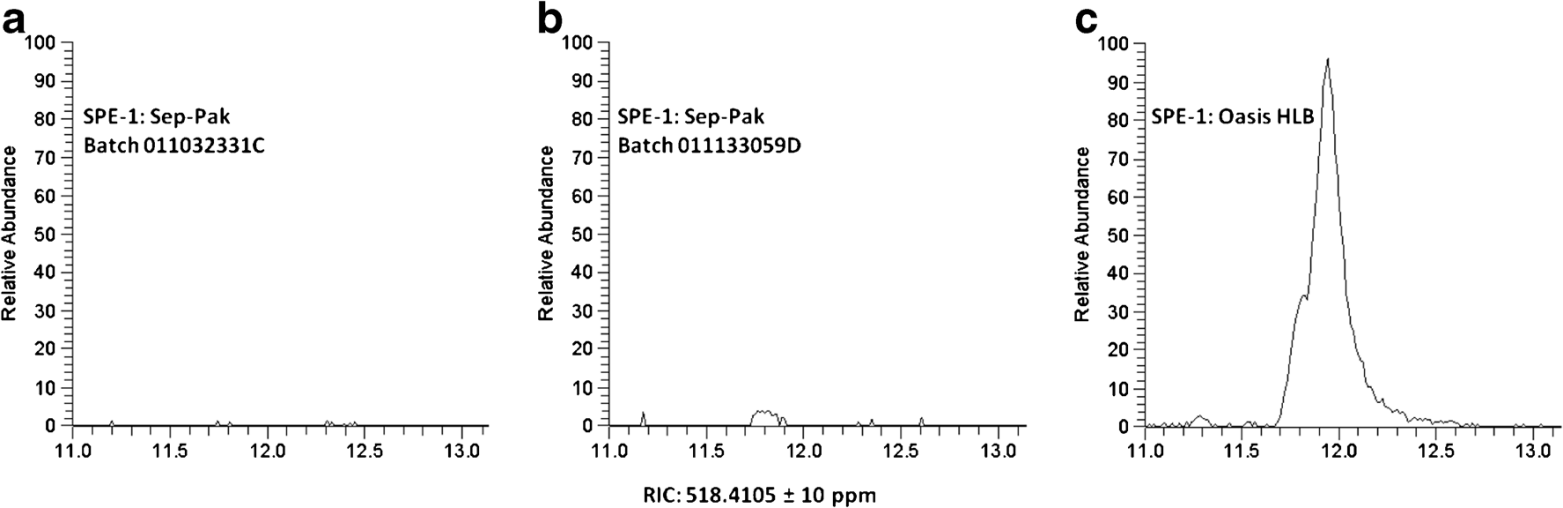
Comparison of SPE cartridges for the removal of cholesterol. Reconstructed ion chromatograms (RIC) for 518.4105 ± 10 ppm. (**a**) Waters Sep-Pak tC18 batch 011032331C, (**b**) Waters Sep-Pak tC18 batch 011133059D, and (**c**) Waters Oasis HLB. Cholesterol retention time: 11.99 min. Waters Oasis HLB was used for SPE-2 in all cases

As the results from our mixture of standards were satisfactory, we next tested the Oasis HLB cartridge using a commercially available plasma sample. The first step of our protocol for extraction of oxysterols from plasma requires the separation of cholesterol from oxysterols by SPE-1 (ESM Fig. S2). Cholesterol is present at levels of about 1000-fold higher than endogenous oxysterols and can generate artefacts by non-enzymatic autoxidation during sample preparation. We have previously found that the Waters Sep-Pak tC18 removes >99.9 % of the cholesterol present in biological samples generating the oxysterol fraction SPE-1-Fr-1. In this study, we found that both batches C and D of Sep-Pak tC18 worked well to remove cholesterol (Fig. 2a, b) but Oasis HLB cartridges were much less effective (Fig. 2c, cholesterol retention time 11.99). C18 cartridges from other manufacturers also worked well for this step (data not shown).

**Fig. 3:**
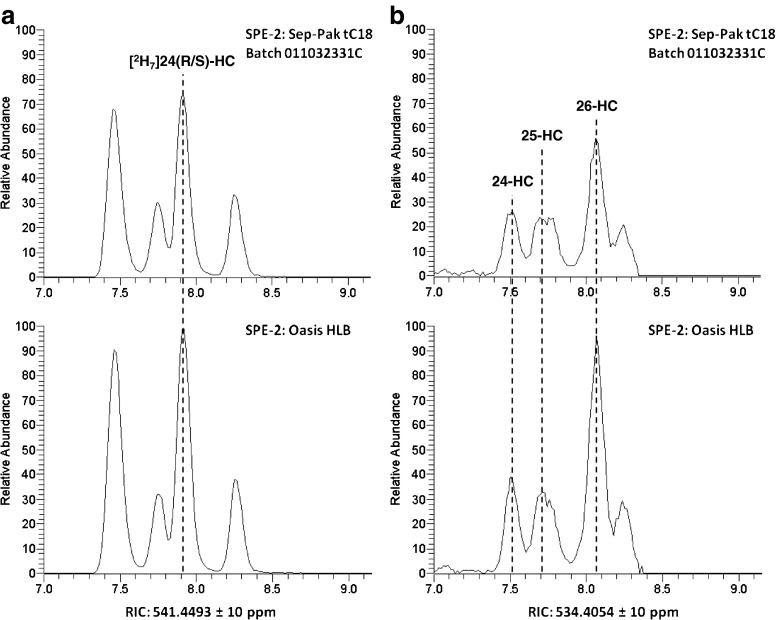
Comparison of SPE cartridges for the recovery of side-chain hydroxycholesterols after EADSA. RICs for (**a**) *m*/*z* 541.4493 ± 10 ppm showing [^2^H_7_]24*R*/*S*-HC. *Top panel* SPE-2 is Waters Sep-Pak tC18 batch 011032331C; *bottom panel* SPE-2 is Waters Oasis HLB and (**b**) RIC for *m*/*z* 534.4054 ± 10 ppm showing endogenous side-chain hydroxycholesterols. *Top panel* SPE-2 is Waters Sep-Pak tC18 batch 011032331C; *bottom panel* SPE-2 is Waters Oasis HLB. Peaks in (**a**) and (**b**) are normalised to the most intense peak in each column

To evaluate the Oasis HLB cartridge for SPE-2, the oxysterol fraction obtained from a Sep-Pak tC18 cartridge (batch C) was split into two identical samples. These were oxidised with cholesterol oxidase and derivatised with the GP reagent in parallel. To remove the excess reagent, we used an Oasis HLB cartridge for one sample and a Sep-Pak tC18 (batch C) for the other. Analysis of the samples by LC-MS^n^ then allowed us to directly compare the two columns. As with the mixture of standards, the two cartridges gave similar peak areas for our internal standard, [^2^H_7_]24*R*/*S*-HC, with a slightly higher signal when using the Oasis HLB sorbent (Fig. 3a). We use this standard to quantify endogenous side-chain hydroxycholesterols, i.e. 24*S*-HC, 25-HC and 26-HC. For these analytes, the peak areas were similar for samples worked up using either of the two cartridges for SPE-2 (Fig. 3b). As well as side-chain hydroxycholesterols, we also analyse oxysterols with the hydroxyl group on the B-ring, i.e. 7α-HC and 7β-HC. For the quantification of these compounds, we add 7α-[25,26,26,27,27,27-^2^H_7_]hydroxycholesterol ([^2^H_7_]7α-HC) to our samples as an internal standard. All of these analytes elute 2–3 min after the side-chain hydroxycholesterols. For these compounds, we observed peak areas approximately three times larger when using the Oasis HLB cartridge compared with the Sep-Pak tC18 cartridge as SPE-2 (ESM Fig. S7). For oxysterols in plasma, we find an intra-batch CV of <15 %. The inter-batch CV for OASIS cartridges is no larger than the intra-batch CV.

## Discussion

Having evaluated a number of SPE cartridges, we have now optimised sample preparation procedure for EADSA. For the separation of cholesterol from oxysterols in SPE-1, C18-based sorbents are the most effective. We use Waters Certified Sep-Pak tC18 columns for this step. This successfully removes >99.9 % of the cholesterol from the oxysterol fraction, whatever batch is used, greatly reducing the risk of generating artefacts by autoxidation. However, as the more recent batches of Sep-Pak tC18 gave unsatisfactory results for the recovery of charge-tagged sterols, we now use Waters Oasis HLB cartridges for SPE-2. In addition, the greater recovery of B-ring hydroxycholesterols using this column is a distinct advantage as it improves the sensitivity and reliability of the method.

## Electronic supplementary material

Below is the link to the electronic supplementary material.

ESM 1 (PDF 200 kb)

## Abbreviations

[^2^H_7_]24R/S-HC: 24*R*/*S*-[25,26,26,26,27,27,27-^2^H_7_]Hydroxycholesterol
26-HC: (25*R*)26-Hydroxycholesterol
7α-HC: 7α-Hydroxycholesterol
BHT: Butylated hydroxytoluene
CYP: Cytochrome P450
DHEAS: Dehydroepiandrosterone sulphate
EADSA: Enzyme-assisted derivatisation for sterol analysis
EBI2: Epstein-Barr virus-induced receptor 2
ESI: Electrospray ionisation
GC: Gas chromatography
GP: Girard P
LC: Liquid chromatography
LXRs: Liver X receptors
MS: Mass spectrometry
MS^n^: Tandem mass spectrometry or mass spectrometry with multistage fragmentation
RIC: Reconstructed ion chromatogram
SPE: Solid-phase extraction
