# Supplementary material for: Revised sample preparation for the analysis of oxysterols by enzyme-assisted derivatisation for sterol analysis (EADSA)
Source: Anal Bioanal Chem. 2015 Mar 22;407(17):5235–9. doi: 10.1007/s00216-015-8609-2 (PMC4471317; doi:10.1007/s00216-015-8609-2)
Supplement: Supplementary file 1 — (PDF 200 kb) [file 216_2015_8609_MOESM1_ESM.pdf]

**Analytical and Bioanalytical Chemistry**

**Electronic Supplementary Material**

# Revised sample preparation for the analysis of oxysterols by enzyme assisted derivatisation for sterol analysis (EADSA)

Peter J. Crick, T. William Bentley, Yuqin Wang, William J. Griffiths

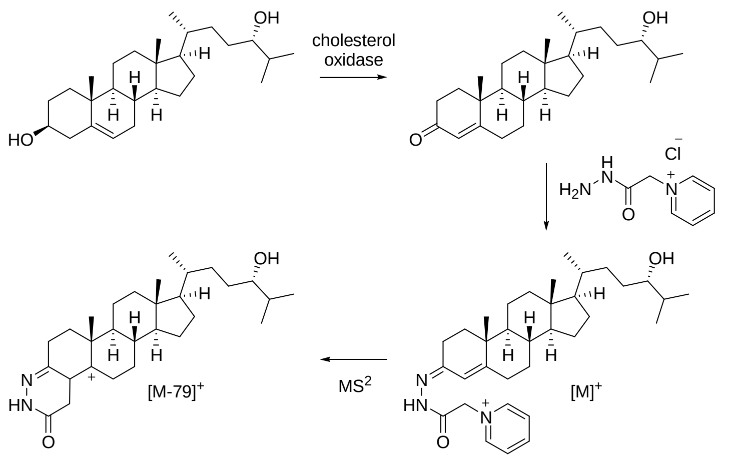


**Fig. S1** EADSA exemplified by the oxidation and derivatisation of 24S-hydroxycholesterol (24S-HC)

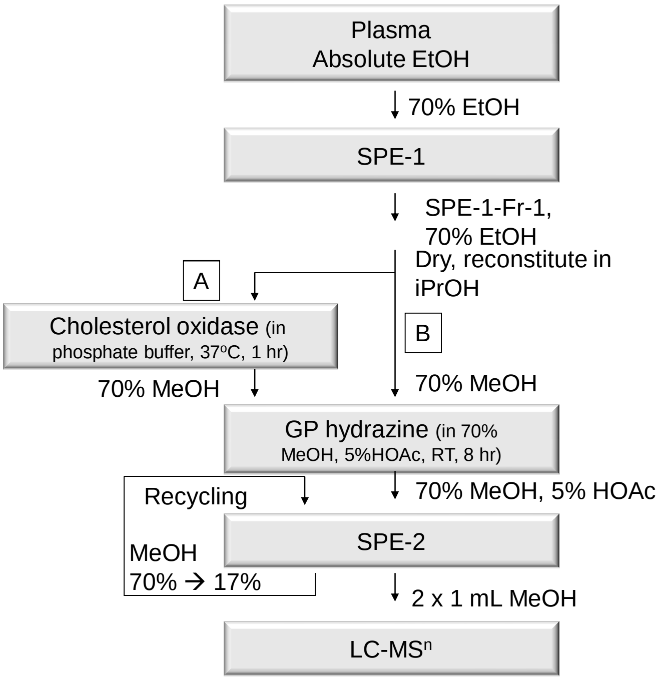


**Fig. S2** EADSA methodology. 100 µL of plasma, or of calibrator, or of plasma mimic, is added to 1.05 mL of ethanol containing deuterated internal standards. The solution is diluted to 70% ethanol and centrifuged. The supernatant (1.5 mL 70% ethanol) is loaded on SPE-1 and the flow-through and a 5.5 mL wash with 70% ethanol combined. This fraction SPE-1-Fr-1, the oxysterol fraction, is dried under reduced pressure, re-constituted in 100 µL of propan-2-ol (iPrOH) and treated with $KH_2PO_4$ buffer (1 mL 50 mM, pH 7) containing 3 µL of cholesterol oxidase (2 mg/mL in $H_2O$, 44 units/mg protein) for 1 hr at 37 $^{o}$C. Methanol (2 mL), glacial acetic acid (150 µL) and GP reagent (150 mg, 0.8 mmole) are added and the mixture incubated at room temperature over night. To remove excess derivatisation agent the reaction mixture is applied to SPE-2. A re-cycling protocol is adopted where the eluate is diluted with an equal volume of water and re-cycled on the column until the eluate is 17.5 % methanol (19 mL). After a wash with 10% methanol (6 mL) GP-derivatised metabolites are eluted in methanol (2 or 3 mL)

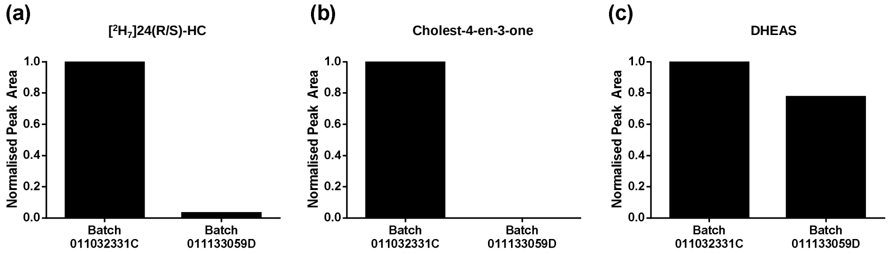


**Fig. S3** Comparison of peak areas from two batches of Sep-Pak tC18 cartridges. (**a**) [$^2H_7$]24R/S-HC; (**b**) cholest-4-en-3-one; and (**c**) DHEAS, after EADSA. Peak areas were normalized to Sep-Pak tC18 batch 011032331C

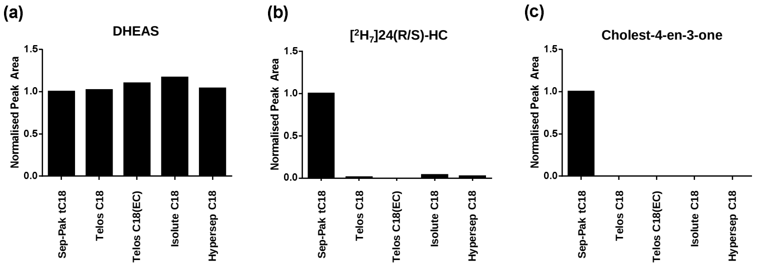


**Fig. S4** Comparison of peak areas obtained after work-up with C18 cartridges from four manufacturers for the analytes, (**a**) DHEAS; (**b**) [$^2H_7$]24R/S-HC; and (**c**) cholest-4-en-3-one after EADSA. Peak areas were normalized to Waters Sep-Pak tC18 batch 011032331C

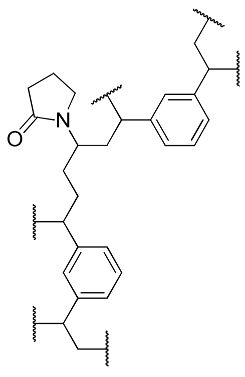


**Fig. S5** Structure of the Oasis HLB polymeric sorbent

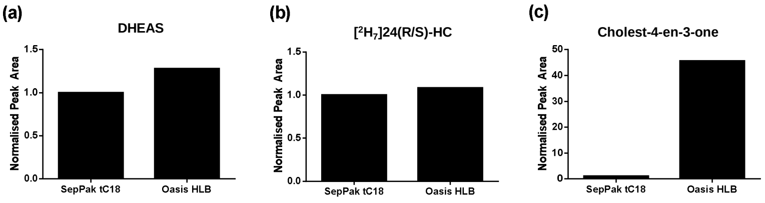


**Fig. S6** Comparison of peak areas obtained after SPE-2 with Waters Sep-Pak tC18 batch 011032331C and Waters Oasis HLB cartridges. (**a**) DHEAS; (**b**) [$^2H_7$]24R/S-HC; and (**c**) cholest-4-en-3-one after EADSA. Peak areas were normalized to Waters Sep-Pak tC18 batch 011032331C

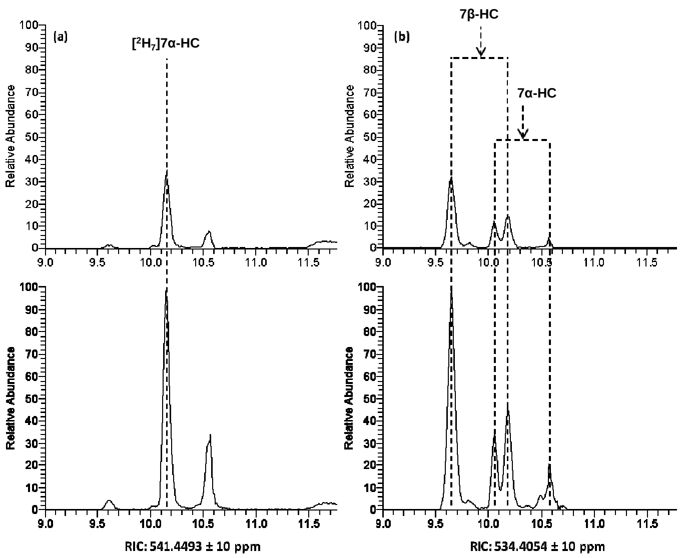


**Fig. S7** Comparison of SPE cartridges for the recovery of B-ring hydroxycholesterols after EADSA. RIC for (**a**) *m/z* 541.4493 ± 10 ppm showing [$^2H_7$]7α-HC. Top panel, SPE-2 is Waters Sep-Pak tC18 batch 011032331C; bottom panel, SPE-2 is Waters Oasis HLB; and (**b**) RIC for *m/z* 534.4054 ± 10 ppm showing endogenous B-ring hydroxycholesterols. Top panel, SPE-2 is Waters Sep-Pak tC18 batch 011032331C; bottom panel, SPE-2 is Waters Oasis HLB. Peaks in (a) and (b) are normalised to the most intense peak in each column. Both 7α-HC and 7β-HC show *syn* and *anti* conformers
